# Loss of LCAT function aggravates metabolic-associated steatohepatitis (MASH) in golden Syrian hamster

**DOI:** 10.1042/CS20257764

**Published:** 2025-11-17

**Authors:** Yuqing Zhang, Huan Wang, Fuhua Wang, Xin Guo, Mingming Zhao, Zihao Zhou, Xiao Lin, Lemin Zheng, Yuhui Wang, George Liu, Guotao Lu, Xunde Xian, Zhao Dong

**Affiliations:** 1 Department of Clinical Diagnosis, Laboratory of Beijing Tiantan Hospital, Capital Medical University, Beijing, China; 2 Institute of Cardiovascular Sciences, School of Basic Medical Sciences, Peking University Health Science Center, Beijing, China; 3 State Key Laboratory of Vascular Homeostasis and Remodeling, Peking University Health Science Center, Beijing, China; 4 Department of Critical Care Medicine, The Affiliated Hospital of Qingdao University, Qingdao, Shandong, China; 5 Department of Cardiology and Institute of Vascular Medicine, Peking University Third Hospital, Beijing, China; 6 State Key Laboratory of Vascular Homeostasis and Remodeling, Institute of Advanced Clinical Medicine; National Health Commission (NHC) Key Laboratory of Cardiovascular Molecular Biology and Regulatory Peptides, Beijing, China; 7 Department of Biomedical Informatics, Center for Noncoding RNA Medicine, State Key Laboratory of Vascular Homeostasis and Remodeling, School of Basic Medical, Peking University Health Science Center, Beijing, China; 8 Pancreatic Center, Department of Gastroenterology, Yangzhou Key Laboratory of Pancreatic Disease, The Affiliated Hospital of Yangzhou University, Yangzhou University, Yangzhou, China; 9 Department of Physiology and Pathophysiology, School of Basic Medical Sciences, Peking University Health Science Center, Beijing, China

**Keywords:** free cholesterol, LCAT, MASH, MASLD, Syrian golden hamster

## Abstract

Lecithin cholesterol acyltransferase (LCAT) plays a pivotal role in acyl-esterifying cholesterol intravascularly, but its function in metabolic dysfunction-associated steatotic liver disease (MASLD) or steatohepatitis (MASH) has remained uncertain both in murine models and humans for decades, which is largely attributable to the distinct differences in cholesterol metabolism between mice and humans. Previously, we created a novel golden Syrian hamster model deficient in LCAT activity. Herein, we explored the influence of LCAT on the development of MASLD and MASH. A cross-sectional clinical study of LCAT activity and free cholesterol (FC) levels in healthy and MASLD patients was performed. LCAT knockout (LCAT KO) hamsters were used to explore the characteristics of cholesterol homeostasis and MASLD and MASH development. Lipidomics, mRNA-seq, and qPCR were employed to investigate the underlying mechanisms involved. MASLD patients displayed reduced LCAT activity, elevated FC levels, and ratio of FC/TC. Serum FC levels were positively correlated with triglyceride (TG), total cholesterol (TC), and apoB100 levels. In hamsters, LCAT deficiency resulted in increased FC levels and decreased high-density lipoprotein levels. Apolipoprotein profiles revealed increased ApoB100/48 and apoE but decreased apoAI. Increases in serum FC levels were primarily observed in LCAT-deficient hamster. Interestingly, LCAT KO hamsters presented mild TG species deposition in the liver even when fed a chow diet indicated by lipidomics. These increased TG species included TG (16:0/18:1/18:2), TG (16:0/18:1/18:3), and TG (16:0/16:1/18:1). On a high-fat and high-cholesterol diet, LCAT-deficient hamsters developed severe liver ballooning, inflammation, and fibrosis. Using HepG2 cells and primary hepatocytes confirmed that FC increased intracellular lipogenesis and promoted inflammatory response, which was reversed by a NLRP3 inhibitor. In summary, LCAT deficiency in hamsters promotes liver lipid deposition and MASH progression, thus highlighting the therapeutic role of LCAT in MASLD and MASH.

## Introduction

Metabolic dysfunction-associated steatotic liver disease (MASLD) is characterized by the accumulation of lipid droplets in hepatocytes and is rapidly becoming the most prevalent liver disease worldwide; MASLD further progresses to metabolic-associated steatohepatitis (MASH), which contributes to chronic metabolic conditions, including insulin resistance, diabetes, cirrhosis, and hepatocellular carcinoma (HCC) [1–3]. In addition to neutral lipids, nonneutral lipids, particularly free cholesterol (FC), are major lipotoxic molecules critical for the development of experimental and human MASLD or MASH [4,5]. Over the past few decades, independent studies have demonstrated that FC can promote steatosis [6], exacerbate inflammation [7] and fibrosis [8], and trigger pyroptosis [9] and endoplasmic reticulum (ER) stress [10] in hepatocytes. However, it remains unclear whether circulating FC homeostasis governs the development of MASLD or MASH.

Lecithin cholesterol acyltransferase (LCAT) is the primary intravascular acyltransferase responsible for reducing FC in circulation by hydrolyzing phosphatidylcholine and then converting FC to CE [11]. LCAT-mutant patients often display lower high-density lipoprotein (HDL) levels, lower CE levels, and elevated FC levels [12,13]. Therefore, LCAT is generally regarded as a protector against metabolic diseases because it promotes reverse cholesterol transport (RCT) and reduces FC [11]. However, the role of LCAT in MASLD and MASH is less clear.

Previous studies have reported conflicting findings showing that LCAT mass is decreased in MASLD [14] and that, in contrast, there is a moderate increase in LCAT activity [15]. Anita M. van den Hoek reported that LCAT expression was down-regulated by approximately 50% in LDLR knockout (LDLR KO) mice with MASLD [16]. However, LCAT deletion did not influence hepatic triglyceride (TG) and FC contents following the consumption of a high-fat, high-sucrose diet [17]. Unlike in LCAT mutant patients, M. Hoekstra and colleagues reported that, in LCAT-deficient mice, plasma FC levels remained unchanged [18]. It is not clear whether the discrepancies between these findings are due to differences in cholesterol metabolism between mice and humans, and the precise role of LCAT in MASLD has not been fully addressed thus far. Further exploration is needed to investigate the role of LCAT in MASLD and MASH.

Hamsters have been shown to have human-like plasma lipid profiles [19]. In humans, circulating total cholesterol (TC, including CE and FC) is associated primarily with low-density lipoprotein (LDL) or very low-density lipoprotein (VLDL) [19]. In contrast, mice exhibit an ‘HDL-predominant’ profile, with the majority of TC, including FC and CE, largely carried in HDL [19]. Unlike those of mice, the cholesterol profiles of hamsters are similar to those of humans, in which 30–40% of TC is distributed on VLDL and LDL [19]. The golden Syrian hamster expresses cholesteryl ester transfer protein (CETP) and only exhibits intestinal apolipoprotein B (apoB) mRNA editing [20]. In contrast, mice lack CETP and have apoB mRNA editing both in the liver and intestine. In our previous work, we developed LCAT KO hamsters via the CRISPR/Cas9 system and reported that their lipid profiles are similar to those of patients with LCAT deficiency. Compared with wildtype (WT) hamsters, LCAT KO hamsters presented threefold increased FC levels [21]. Thus, the LCAT KO hamster is an optimized rodent model to elucidate the function of LCAT in MASLD and MASH.

In this study, we enrolled MASLD patients and found that LCAT activity was lower and the plasma FC level was higher in MASLD patients than in healthy controls. Next, we fed LCAT KO hamsters either a chow diet or a high-fat and high-cholesterol (HFHC) diet. The lipidomic results revealed that LCAT deficiency mildly promoted liver TG deposition in LCAT KO hamsters on a chow diet. Upon HFHC diet feeding, LCAT deficiency in hamsters exacerbated MASH, with increased hepatic ballooning, inflammation, and fibrosis, suggesting that LCAT plays a protective role against MASH and could be a therapeutic target for treating MASLD or MASH.

## Materials and methods

### Human study

In this cross-sectional study, 79 MASLD patients and 59 healthy controls from the Affiliated Hospital of Qingdao University, Qingdao, Shandong, China, were recruited to measure serum FC levels and LCAT activity. MASLD patients were diagnosed by ultrasound detection as previously described [22] who had complete information on demographics, personal characteristics (age, sex, weight, height, and BMI), and clinical characteristics (blood glucose, lipid concentrations, liver functions, urea nitrogen, and serum creatinine). Healthy controls were free from metabolic and vascular disease such as diabetes, hypertension, coronary artery disease, and cerebrovascular disease. The age and gender in healthy controls were matched to those in MASLD. The exclusion criteria included pulmonary, renal, and pancreatic diseases; a history of alcohol abuse; and other known liver diseases.

### Assays of human serum biochemical parameters

Serum was isolated by centrifuging blood at 3000 rpm for 10 minutes at 4°C. Serum levels of alanine aminotransferase (ALT), aspartate aminotransferase (AST), γ-glutamyl transpeptidase (GGT), alkaline phosphatase (ALP), total protein (TP), albumin (ALB), globulin (GLB), total bilirubin (TBIL), direct bilirubin (DBIL), total bile acid (TBA), lactate dehydrogenase (LDH), creatine kinase (CK), hydroxybutyrate dehydrogenase (HBDH), glucose (Glu), urea, creatinine (Cr), uric acid (UA), TG, TC, HDL cholesterol (HDL-C), low-density lipoprotein cholesterol (LDL-C), apolipoprotein A1 (apo A1), apolipoprotein B100 (apo B100), homocysteine (Hcy), and creatine kinase MB (CK-Mb) were measured using an Olympus AU 2700 analyzer (Olympus, Tokyo, Japan) in accordance with the manufacturer’s instructions.

### Hamster study

The LCAT KO Syrian golden hamster model was generated using CRISPR/Cas9 technology in our laboratory as described previously [21]. Briefly, sgRNA was designed to target exon 2 of the *Lcat* gene (NW_004801665). Cas9 mRNA and sgRNA were coinjected into the cytoplasm of zygotes. Genotyping to confirm the presence of the mutation was conducted via PCR using the following primers: F: ACCAGGAGCATTGGACACG, R: GGGAATCAGCTGGAAGCCAA. In our experiments, LCAT KO hamsters and WT hamsters were matched by age and sex, with all the animals being 8–12 weeks old. To induce MASLD, eight-week-old hamsters (LCAT KO and WT) weighing 110–130 g were selected for the experiment. All the animals were fed either a chow diet or a HFHC diet (0.5% cholesterol and 10% lard, w/w, based on a powdered chow diet) provided by BiotechHD Co. Ltd., Beijing, China, for 12 weeks. Water was available ad libitum. At the end of experiments, isoflurane was used for killing the animals. Hamsters were inhaled 0.41 ml/min at 4 L/min fresh gas flow (approximate 2%) of isoflurane with careful monitoring to avoid pain or discomfort. Blood was collected from the heart. After perfusion with PBS, the liver was collected for further analysis.

All of the hamsters were housed under the standard conditions with 23°C±2°C, a humidity level of 50%-60%, and a 12/12 hours light–dark cycle at the Department of Laboratory Animal Science of Peking University Health Science Center. All animal procedures were performed in accordance with the guidelines of Laboratory Animal Care (NIH publication no. 85–23, revised 1996) and were approved by the Animal Care and Use Committee of the Peking University Health Science Center.

### Determination of the phospholipase activity of LCAT

Phospholipase activity of LCAT was measured using a commercial kit (Sigma, MAK107-1KT). The fluorometric substrates were incubated with 4 μl of plasma at 37°C for 2.5 h. Heat-inactivated plasma was used as a negative control to eliminate endogenous autofluorescence interference. The emission spectrum of the substrate reagent showed two distinct peaks at 390 nm and 470 nm. After incubation, LCAT activity was evaluated as the ratio of the change in fluorescence intensity at 390 nm to that at 470 nm after excluding the value for endogenous autofluorescence.

### Determination of cholesterol esterification rate of LCAT

Cholesterol esterification rate of LCAT was determined by modified previous study [23]. In brief, the esterification rate of LCAT was determined by measuring the decrease in the concentration of endogenous substrate (plasma FC levels) during incubation of whole plasma at 37°C for 4 h. The cholesterol esterification rate of LCAT (μmol/L/hour) = [(initial plasma FC) – (final plasma FC)]/incubation time (4 h)

### Measurement of LCAT mass via ELISA

LCAT mass was detected by commercial ELISA (Catalog: SEJ516HU, Cloudy-Clone, Wuhan, China), according to the manufacturer’s instructions. Briefly, a sandwich enzyme immunoassay was used for the *in vitro* quantitative measurement of LCAT in human serum. Human serum was diluted 1000-fold using PBS, and 100 μl sample was added to anti-LCAT antibody coated 96-well plate. After incubation at 37°C for 1 h, primary anti-LCAT antibody was added for another incubation. After three washes, the HRP-labeled secondary anti-IgG antibody was added. Finally, after five washes, the TMB Substrate was added and incubated for 10 min. The STOP solution was immediately added for measurement at 450 nm. The LCAT concentration was calculated using a standard curve.

### Plasma lipid and lipoprotein analysis

Blood samples were collected from hamsters into heparinized capillary tubes after an overnight fast, and plasma was isolated by centrifugation (3000 rpm, 4°C, 10 min). TC and TG concentrations were measured using commercial kits (TR0100, MAK043, Sigma‒Aldrich, USA). The FC content was measured using a commercial kit from Applygen (E1006, Applygen, Beijing, China).

Plasma lipoprotein fractions were separated and collected using an ÄKTA fast protein liquid chromatography (FPLC) system (Amersham Biosciences). Briefly, pooled plasma from 8 aliquots from each group was processed, and 100 μl was eluted with buffer through a Superose 6 HR 10/300 column (GE) at a constant flow rate of 0.5 ml/min. Fractions (500 μl) were collected for the measurement of TG, TC, and FC contents.

### Apolipoprotein analysis

Hamsters were fasted overnight, and then, blood was collected into a heparin-containing tube. Plasma was isolated by centrifuging the blood samples at 3000 rpm and 4°C for 10 min. Lipoproteins were isolated by ultracentrifugation at 60,000 rpm and 10°C for 12 h at a density of >1.21 g/ml. The isolated lipoproteins were mixed with methanol and diethyl ether (v/v: sample: methanol: diethyl ether = 25 μl:1.5 ml:3.5 ml) and vortexed for 1 min to remove lipids. After centrifugation at 1000 rpm and 4°C for 4 min, the sample was dried under nitrogen. SDS‒PAGE was then performed, and Coomassie blue staining of the gel was used for apolipoprotein analysis.

### Culture and stimulation of the HepG2 cell line and primary hamster hepatocyte

Human hepatoma HepG2 cells (ATCC, Manassas, VA) were cultured in Dulbecco’s modified Eagle’s medium (DMEM; Gibco, Paisley, Scotland, U.K.) supplemented with 10% fetal bovine serum (FBS; HyClone, Victoria, Australia) in a CO_2_ incubator at 37°C. Hamster primary hepatocytes were isolated using collagenase IV and purified by centrifugation. In brief, first, fresh liver was perfused by D-hank’s buffer supplemented with 500 μmol/L EGTA, without Ca^2+^ and Mg^2+^, via hepatic portal vein. And then, liver was perfused by D-hank’s buffer supplemented with collagenase IV and 2 mmol/L CaCl_2_. After digestion, the hepatocytes were passed through a 70 μm cell strainer and washed by cold PBS three times via 4°C, 50 g, 2 min centrifuge. The fresh hepatocytes were resuspended and seeded in six-well attachment plates, and the medium was replaced with fresh RPMI 1640 containing 10% FBS. After 6  h incubation and adherence, cells were administered with different treatments. Palmitic acid (PA) was dissolved in isopropanol at a concentration of 40 mM as a stock solution. The final concentration of PA in DMEM (containing 0% FBS and 0.5% BSA) was 400 µM. The various doses of FC treated HepG2 cells or primary hamster hepatocytes along with PA for 24 h to evaluate the changes of lipogenesis.

### mRNA sequencing

Total RNA was sent to Novogene Co., Ltd. (Beijing, China) for transcriptome analysis. In brief, the quality and quantity of the purified RNA were assessed using a NanoDrop spectrophotometer (Thermo Fisher Scientific, Waltham, MA, U.S.A.) and agarose gel electrophoresis. The RNA integrity number (RIN) ranged from 8.60 to 9.50 in liver tissues from WT and KO hamsters (Supplementary Table S3). All samples were sequenced using a NovaSeq 6000 sequencer (Illumina, San Diego, CA, U.S.A.). The error rate in mRNA sequence increased from 0.02% to approximately 0.035% per 150 reads as shown in Supplementary FigureS4. After clean data were obtained, analyses were performed using Novomagic (https://magic.novogene.com/customer/main#/homeNew). The mRNA-expressing profiles reported in this paper have been deposited in the OMIX, China National Center for Bioinformation / Beijing Institute of Genomics, Chinese Academy of Sciences (https://ngdc.cncb.ac.cn/omix: accession Accession: PRJCA043111 [24], OMIX010979 [25]). The expression patterns of genes whose expression significantly differed were analyzed by clustering analysis. Gene Ontology (GO) analysis, Kyoto Encyclopedia of Genes and Genomes (KEGG) analysis, and gene set enrichment analysis (GSEA) were performed to screen for differentially expressed genes. Significance was set at *P*<0.05 and |fold change| > 2.

### Analysis of lipid content in liver, HepG2 cells, or primary hamster hepatocytes

Approximately 100 mg of liver tissue was homogenized in 1 ml of PBS. For HepG2 cells and primary hamster hepatocytes, 1 × 10^6^ cells were homogenized and ultrasonicated in 100 μl of PBS. Lipids were extracted using the method described by Folch et al. [26] and dissolved in 500 μl of 3% Triton X-100 for liver tissue and 20 μl of 3% Triton X-100 for HepG2 cells. TG or TC levels were measured using enzymatic methods, as described earlier.

### Lipidomic analysis of the liver

Lipids were extracted from liver samples using a modified Bligh–Dyer method [27]. Briefly, 100 mg of liver tissue was homogenized in 1 ml of cold PBS. Lipids were extracted by adding chloroform/methanol (v:v = 2:1). The samples were vortexed for 2 min, incubated for 20 min, and then centrifuged at 1000 rpm for 5 min. The lower chloroform layer was collected and dried under nitrogen. LC/MS was used for the lipidomic analysis. Reverse-phase chromatography was performed using a Cortecs C18 column (2.1 × 100 mm, Waters). Mobile phase A comprised 400 ml of HPLC-grade water containing 0.77 g of ammonium acetate mixed with 600 ml of HPLC-grade acetonitrile (pH~7). Mobile phase B comprised 10% acetonitrile (ACN) and 90% isopropanol (IPA) (v/v). For mass spectrometry, data were acquired using a QExactive Orbitrap mass spectrometer (Thermo, CA) coupled with an Ultimate 3000 UHPLC system (Thermo, CA). For the data analysis, lipids were identified and quantified using LipidSearch software v4.1.16 (Thermo, CA). LipidSearch v4.1.16 allows lipid identification on the basis of MS/MS matching [28]. All lipid classes in the database were chosen for identification. Adducts of −H and +CH3 COO were selected for negative mode because of the use of ammonium acetate in the mobile phases [29].

### RNA isolation and quantitative real-time PCR

Total RNA was extracted from tissue using TRIzol reagent (Invitrogen, Carlsbad, CA). First-strand cDNA was synthesized using a reverse transcription (RT) kit (Invitrogen). Quantitative real-time PCR (qRT‒PCR) was performed using the primers listed in Supplementary Table S1. All samples were quantified using the comparative CT method for relative quantitation and normalized to *beta-actin*.

### Histological studies

The liver was sectioned (thickness, 7 μm) for Oil Red O and BODIPY staining using a cryostat. Paraffin-embedded sections were stained with hematoxylin and eosin (H&E) or Sirius Red to assess fibrosis. Immunohistochemical staining of IL-1β (ab283818, Abcam, U.S.A.) was used to assess inflammation. NAFLD activity scores (NASs) summarizing the main histological lesions were determined on the basis of the steatosis grade and activity grade (hepatocyte ballooning and lobular inflammation) (Supplementary Table S2), as previously described [30].

### Western blot

Tissues or cells were harvested and lysed in RIPA buffer (C1053-500, APPLYGEN, China) supplemented with protease inhibitor (04693132001, Roche, Switzerland) and phosphatase inhibitor (4906845001, Roche, Switzerland). The supernatants were collected and protein concentration was measured by Pierce BCA Protein Assay (23225, Thermo Fisher, U.S.A.). Protein lysates were heated in 1X loading buffer at 37°C for 1  h. A 20–30 µg of proteins was loaded in SDS-PAGE, and then transferred to nitrocellulose (NC) membrane. Primary antibodies: NLRP3 (TB4673, Abmart, China), IL1-beta (12242, Cell Signaling, U.S.A.), TGF-beta (A25313, Abclonal, China), COL-1 (14695–1-AP, Proteintech, China), GAPDH (60004–1-Ig, Proteintech, China) were indicated overnight. After washing with 1X TBST, the second antibodies were added for incubation for another 1  h. The target protein bands were visualized with enhanced chemiluminescence solution (36,208ES, Yeasen, China) using the iBright lmaging System (CL1500, Invitrogen, U.S.A.).

### Quantification and statistical analysis

The data are presented as means ± standard errors of the means (SEMs). Numbers refer to the biological replicates in independent studies. All the statistical analyses were performed using GraphPad Prism 10.0 software (GraphPad Software, San Diego, CA, U.S.A.). The distribution of the data was first evaluated using the Shapiro‒Wilk test. Differences between two groups were compared using Student’s *t*-test for normally distributed data and the Mann‒Whitney test for nonnormally distributed data. One-way ANOVA with Tukey’s test for normally distributed data and one-way ANOVA with the Kruskal‒Wallis test for nonnormally distributed data were used to analyze differences among three or more groups. The detailed statistical analysis applied to each experiment is presented in the corresponding figure legends. A value of *P*<0.05 was considered statistically significant. Representative images were chosen to most accurately represent the group mean/average across all the available data.

## Results

### The activity of LCAT is impaired and the serum FC level is increased in MASLD patients

**Figure 1:**
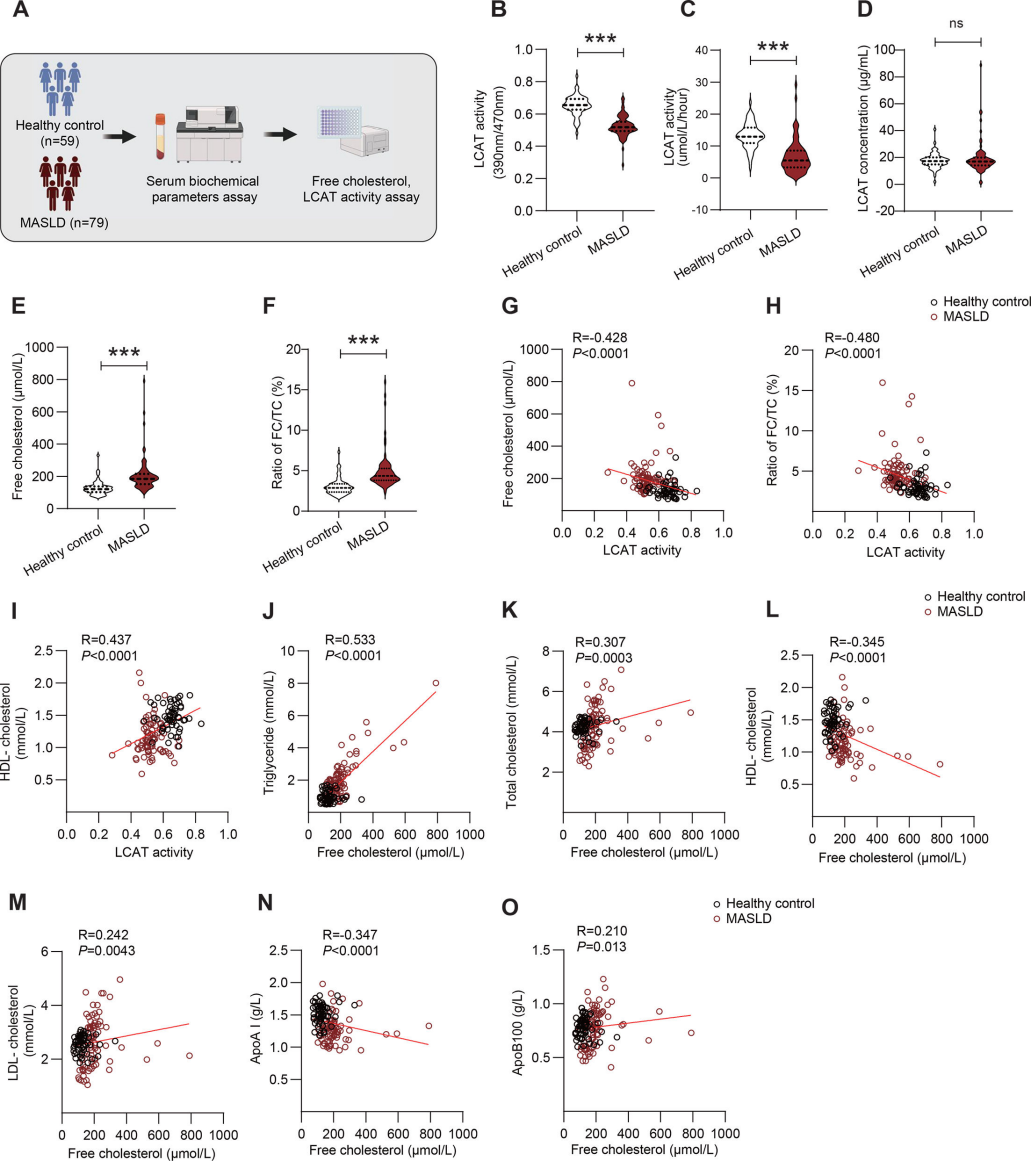
**LCAT activity is reduced and serum free cholesterol is increased in MASLD patients**. (**A**) Schematic workflow of the cross-sectional clinical study design. (**B-D**) Comparison of serum phosphatase of LCAT activity (**B**), cholesterol esterified rate of LCAT activity (**C**) and LCAT concentration (**D**) between healthy controls (*n* = 59) and MASLD patients (*n* = 79). (**E-F**) Comparison of serum free cholesterol (**E**) and ratio of FC/TC (**F**) between healthy controls (*n* = 59) and MASLD patients (*n* = 79). (**G-I**) Correlation analysis showing the relationships between LCAT activity and free cholesterol (**G**), ratio of FC/TC (**H**) and HDL-cholesterol (**I**) (Healthy controls (*n* = 59), MASLD patients (*n* = 79)). (**J-O**) Correlation analysis showing the relationships between free cholesterol and triglycerides (**J**), total cholesterol (**K**), HDL cholesterol (**L**), LDL cholesterol (**M**), apolipoprotein A-I (**N**), and apolipoprotein B100 (**O**) (healthy controls (*n* = 59) and MASLD patients (*n* = 79)). Student’s *t*-test was used in panel B. The Mann‒Whitney test was used in panels C, D, E, and F. Spearman correlation analysis was employed in panels G-O. *P<0.05; **P<0.01; and ***P<0.001. HLD, high-density lipoprotein; LDL, low-density lipoprotein; MASLD, metabolic dysfunction-associated fatty liver disease.

Given that the role of LCAT in MASLD is not yet fully understood, herein, 138 subjects were enrolled in a cross-sectional clinical study to investigate the changes in LCAT activity and FC in MASLD. Among the subjects, 79 were diagnosed with MASLD via ultrasound detection, and 59 were healthy controls (Figure 1A). The mean age of the MASLD patients (57.73 ± 11.69) was not significantly different from that of the healthy subjects (55.92 ± 5.46), and the gender of healthy controls was matched to MASLD patients. Among the risk factors for MASLD, serum TG was increased in MASLD patients (healthy controls vs. MASLD patients: 0.95 ± 0.34 vs. 2.12 ± 1.22 mmol/L, *P*<0.0001; Table 1). HDL-C was moderately reduced in MASLD patients (healthy controls *vs*. MASLD patients: 1.48 ± 0.19 vs. 1.15 ± 0.27 mmol/L, *P*<0.0001; Table 1). Fasting blood glucose (FBG) in MASLD patients was elevated but below the diabetes diagnosis threshold (FBG<7.0 mmol/L) (healthy controls vs. MASLD patients: 5.06 ± 0.40 vs. 6.66±2.31 mmol/L, *P*<0.0001; Table 1). The alanine transaminase (ALT) level was mildly elevated in MASLD patients (healthy controls vs. MASLD patients: 19.54 ± 7.74 vs. 25.79±20.31 U/L, *P*=0.04; Table 1), but both were below the clinical cutoff value of 40 U/L. Aspartate transaminase (AST) levels did not differ between the two groups. The kidney function parameters (such as urea nitrogen and creatinine) were not different between MASLD patients and healthy controls. Further detailed clinical characteristics are provided in Table 1. LCAT is considered a vital acyltransferase for the formation of cholesterol esters to reduce FC. Serum LCAT activity was assessed via a fluorometric assay to investigate substrate transfer. We found that phospholipase activity of LCAT was significantly decreased in MASLD patients (healthy controls *vs*. MASLD patients: 0.652 ± 0.008 vs. 0.542 ± 0.007, *P*<0.0001; Figure 1B). Meanwhile, cholesterol esterification rate of LCAT was assayed by the decrease of endogenous FC levels, patients with MASLD displayed lower cholesterol esterification rate than healthy control (13.21 ± 0.511 vs. 6.72 ± 0.583 μmol/L/h, *P*<0.0001, Figure 1C). Serum LCAT concentrations in patients with MASLD did not differ from healthy controls (Figure 1D). Moreover, both serum FC levels and FC/TC ratio were enhanced in MASLD patients than in healthy controls (Figure 1E and F). In line with previous studies, LCAT activity was negatively correlated with serum FC levels and FC/TC ratio and positively correlated with serum HDL levels (Figure 1G, H1). Serum TG and TC disorders are often regarded as risk factors for MASLD. Herein, to elucidate the relationships between FC and these well-known lipid risk factors, we performed Spearman correlation analysis. The results revealed that FC was positively correlated with TG, TC, LDL-C, and apoB100 (Figure 1J, K, M and O) but negatively correlated with HDL-C and apo AI (Figure 1L and N). We have previously reported that hypertriglyceridemia occurred in LCAT KO hamsters. Here, we found that LCAT activity was negatively correlated with serum TG levels in patients’ serum (Supplementary Figure S1A). Unexpectedly, we also found that FC was positively correlated with alkaline phosphatase (ALP) and γ-glutamyl transpeptidase (GGT) and negatively correlated with total bilirubin (TBIL) and direct bilirubin (DBIL), probably indicating changes in hepatic and cholecystic functions (Supplementary Figure S1B-E). In other clinical parameters, FC exhibited correlations with liver protein metabolism, showing a negative correlation with total protein and albumin, but positive correlations with globulin. In terms of kidney functions, FC level was positively correlated with UA, but there were no significant correlations between FC level and other parameters, including urea, Cr, and eGFR. Additionally, FC displayed a positive correlation with fasting blood glucose, CK, and CK-Mb. However, the changes in FC content were independent of the subjects’ age. The detailed correlation matrix is shown in the supplemental materials (Supplementary Figure S1F). Finally, to exclude the effects of statin on FC or LCAT activity, we analyzed plasma TC, FC, and LCAT activity in MASLD patients with (*n* = 32) and without statin administration (*n* = 47). The results showed decreased serum TC levels in MASLD patients with statin therapy compared with those without statin (Supplementary Figure S1H). However, LCAT activity and FC levels in MASLD patients with statin therapy did not differ from those without statin therapy (Supplementary Figure S1Iand G). Taken together, these findings suggest that LCAT may play a potential role in the pathogenesis of MASLD.

**Table 1:** Clinical characteristics of healthy controls and MASLD patients

	Healthy controls	MASLD	*P*-value
Characteristics	N = 59	N = 79	
Demographics			
Age (y)	55.92 ± 5.46	57.11 ± 11.17	0.440
Gender (male) — n (%)	38 (64.41)	52 (65.82)	0.857^a^
BMI (kg/m^2^)	24.14 ± 1.87	26.73 ± 2.90	＜0.0001*
Medical history — n. (%)			
Diabetes	0(0)	41 (51.90)	
Hypertension	0(0)	56 (70.89)	
Coronary artery disease	0(0)	34 (43.04)	
Cerebrovascular disease	0(0)	26 (32.91)	
Statin used	0(0)	32 (40.51)	
Laboratory results			
Alanine transaminase, ALT (U/L)	19.54 ± 7.74	25.79 ± 20.31	0.040*
Aspartate transaminase, AST (U/L)	20.30 ± 4.74	20.93 ± 10.45	0.780
γ-Glutamyl transpeptidase, GGT (U/L)	20.29 ± 9.43	42.47 ± 49.61	0.001*
Alkaline phosphatase, ALP (U/L)	66.53 ± 12.77	73.20 ± 20.81	0.042*
Total protein (g/L)	71.25 ± 2.75	66.33 ± 5.23	＜0.0001*
Albumin (g/L)	44.54 ± 2.06	39.90 ± 2.86	＜0.0001*
Total bilirubin (μmol/L)	12.50 ± 3.03	11.03 ± 4.41	0.027
Direct bilirubin (μmol/L)	3.34 ± 0.97	2.94 ± 1.42	0.066
Total bile acid (μmol/L)	3.03 ± 1.90	4.42 ± 3.58	0.008*
Glucose (mmol/L)	5.06 ± 0.40	6.66 ± 2.31	<0.0001*
Urea nitrogen (mmol/L)	5.92 ± 6.06	5.50 ± 1.35	0.555
Creatinine (μmol/L)	62.22 ± 9.41	62.38 ± 14.12	0.941
Total triglyceride (mmol/L)	0.95 ± 0.34	2.12 ± 1.22	<0.0001*
Total cholesterol (mmol/L)	4.24 ± 0.33	4.27 ± 1.03	0.907
Free cholesterol (μmol/L)	128.36 ± 43.78	197.85 ± 101.88	<0.0001*
FC/TC ratio (%)	3.038 ± 1.026	4.941 ± 2.29	<0.0001*
High density lipoprotein cholesterol, HDL-C (mmol/L)	1.48 ± 0.19	1.15 ± 0.27	＜0.0001*
Low-density lipoprotein cholesterol, LDL-C (mmol/L)	2.58 ± 0.31	2.63 ± 0.90	0.740
Apolipoprotein a1, apo A1 (g/L)	1.52 ± 0.15	1.28 ± 0.20	＜0.0001*
Apolipoprotein b100, apo B100 (g/L)	0.78 ± 0.09	0.77 ± 0.17	0.564

Data were presented as mean ± standard deviation (SD). Student’s *t*-test was used to compare MASLD with Healthy controls. If the data were not a normal distribution, Mann–Whitney U test was used. a,Chi-square test was used to compare MASLD with healthy controls.

### LCAT deficiency elicits an increased plasma FC in golden Syrian hamsters

**Figure 2:**
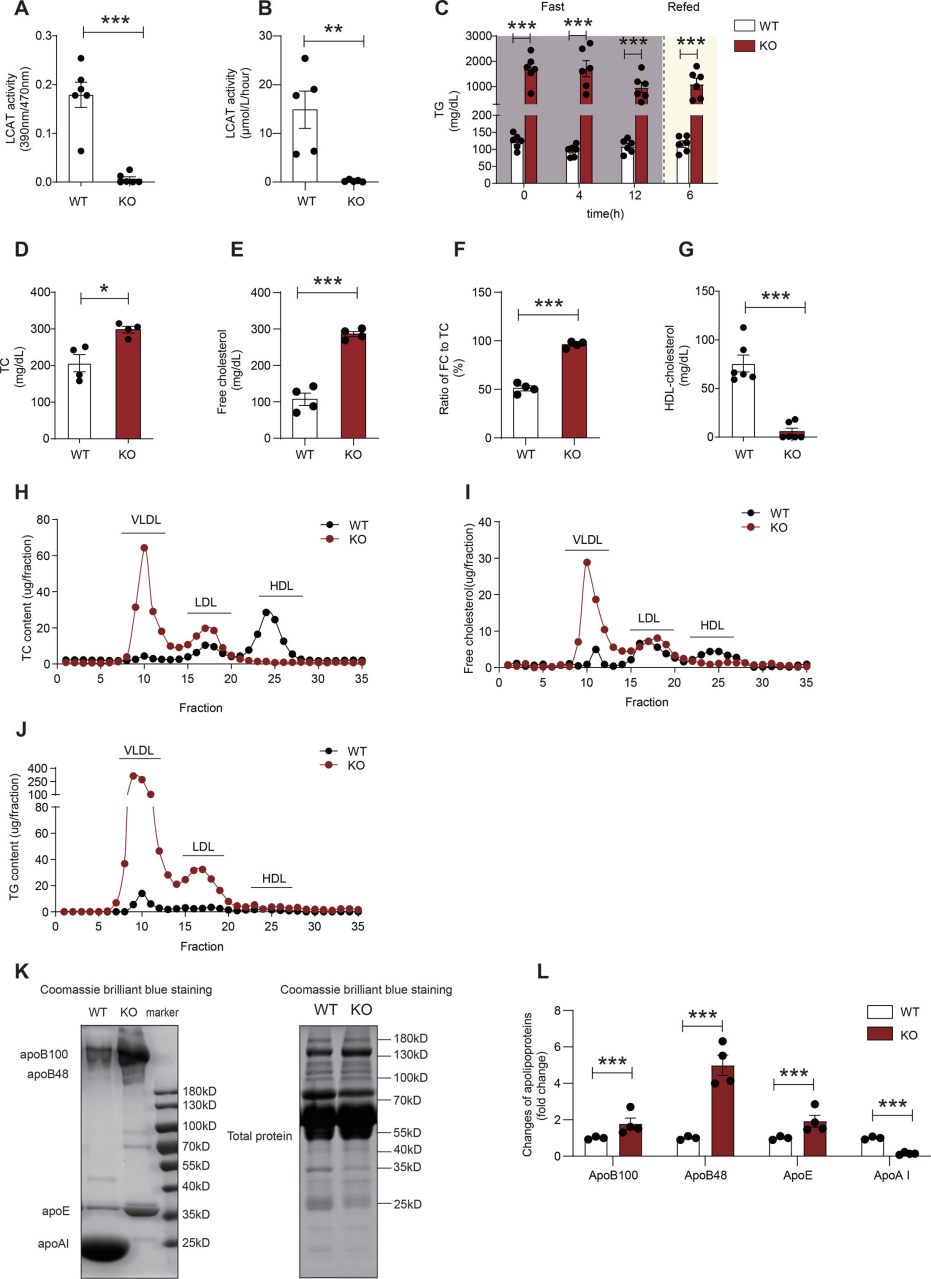
LCAT deficiency increases the plasma free cholesterol level. (**A-B**) Changes in phosphatase activity of LCAT (**A**) and cholesterol esterified rate activity of LCAT (**B**) in LCAT knockout (KO) and wildtype (WT) hamsters (*n* = 5–6/group). (**C**) Time course of fasted and refed plasma triglyceride levels in LCAT KO and WT hamsters following 4 h and 12 h fasting periods, followed by 6 h of refeeding (*n* = 6/group). (**D-G**) Changes in plasma total cholesterol (**D**), free cholesterol (**E**), the ratio of FC to TC (**F**) (*n* = 4/group) and HDL cholesterol (**G**) (*n* = 6/group) in WT and LCAT KO hamsters. (**H-J**) Lipoprotein profile analysis via FPLC of pooled plasma samples (each pool derived from eight individuals) tested for total cholesterol (**H**), free cholesterol (**I**), and triglyceride (**J**) from WT and LCAT KO hamsters. (**K-L**) The distribution of plasma apolipoproteins (apoB-100, apoB48, apoE, and apoA-I) analyzed by SDS‒PAGE with Coomassie blue staining from WT and LCAT KO hamsters (**K**) and quantitative histograms (right panel) (**L**) (*n* = 3–4/group). Student’s *t*-test was used in panels A, B, C, D, E, F, G, and L. *P<0.05; **P<0.01; and ***P<0.001. apoAI, apolipoprotein AI; apoB100, apolipoprotein B100; apoB48, apolipoprotein B48; apoE, apolipoprotein E;TC, total cholesterol; TG, triglyceride.

To address the above cholesterol disorders in MASLD patients, we employed LCAT KO hamsters for subsequent experiments. In LCAT KO hamsters, both phospholipase and cholesterol esterification rate of LCAT activity was completely absent (Figure 2A and B). Compared with WT hamsters, LCAT KO hamsters on a chow diet presented hypertriglyceridemia, but the difference between fasted and refed states was not statistically significant (Figure 2C). Previous studies have demonstrated that this hypertriglyceridemia is likely due to the inhibition of lipoprotein lipase (LPL) activity and the overproduction of VLDL [21]. Herein, we revealed that this hypertriglyceridemia could be partially reversed by fenofibrate treatment in LCAT KO hamsters (Supplementary Figure S2A). Unlike those in mice, TC levels were moderately higher in LCAT KO hamsters than in WT hamsters (Figure 2D). The plasma FC levels were three-fold higher in LCAT KO hamsters than in WT hamsters (Figure 2E). The FC to TC ratio was nearly 100% in LCAT KO hamsters and 50% in WT hamsters (Figure 2F). Additionally, HDL-C was almost undetectable (Figure 2G). Fasting plasma glucose remained unaffected (Supplementary Figure S2B). These data indicate that the CE fraction was almost erased and that the major cholesterol species in LCAT KO hamsters was FC. To determine which lipoprotein fraction was associated with increased FC, the lipoprotein fractions were separated by fast protein liquid chromatography (FPLC). We found that HDL fractions were eliminated, whereas VLDL and LDL fractions were increased, indicated by TC tests in LCAT KO hamsters (Figure 2H). Elevated TG content was assembled in VLDL and LDL particles (Figure 2J). Interestingly, FC content in VLDL components was significantly increased (Figure 2I). Furthermore, all lipoproteins in the plasma were isolated by ultracentrifugation (60,000 rpm for 12 h at a density > 1.21 g/ml) and analyzed via SDS‒PAGE with Coomassie blue staining. As expected, the levels of apoB100, apoB48, and apoE increased, whereas the level of apoA-I almost vanished (Figure 2K and L). Collectively, these data clearly demonstrate that LCAT deficiency leads to both FC and FC/TC disorders in Golden Syrian hamsters.

### LCAT deficiency elicits mild hepatic TG species deposition in golden Syrian hamsters fed a chow diet

**Figure 3:**
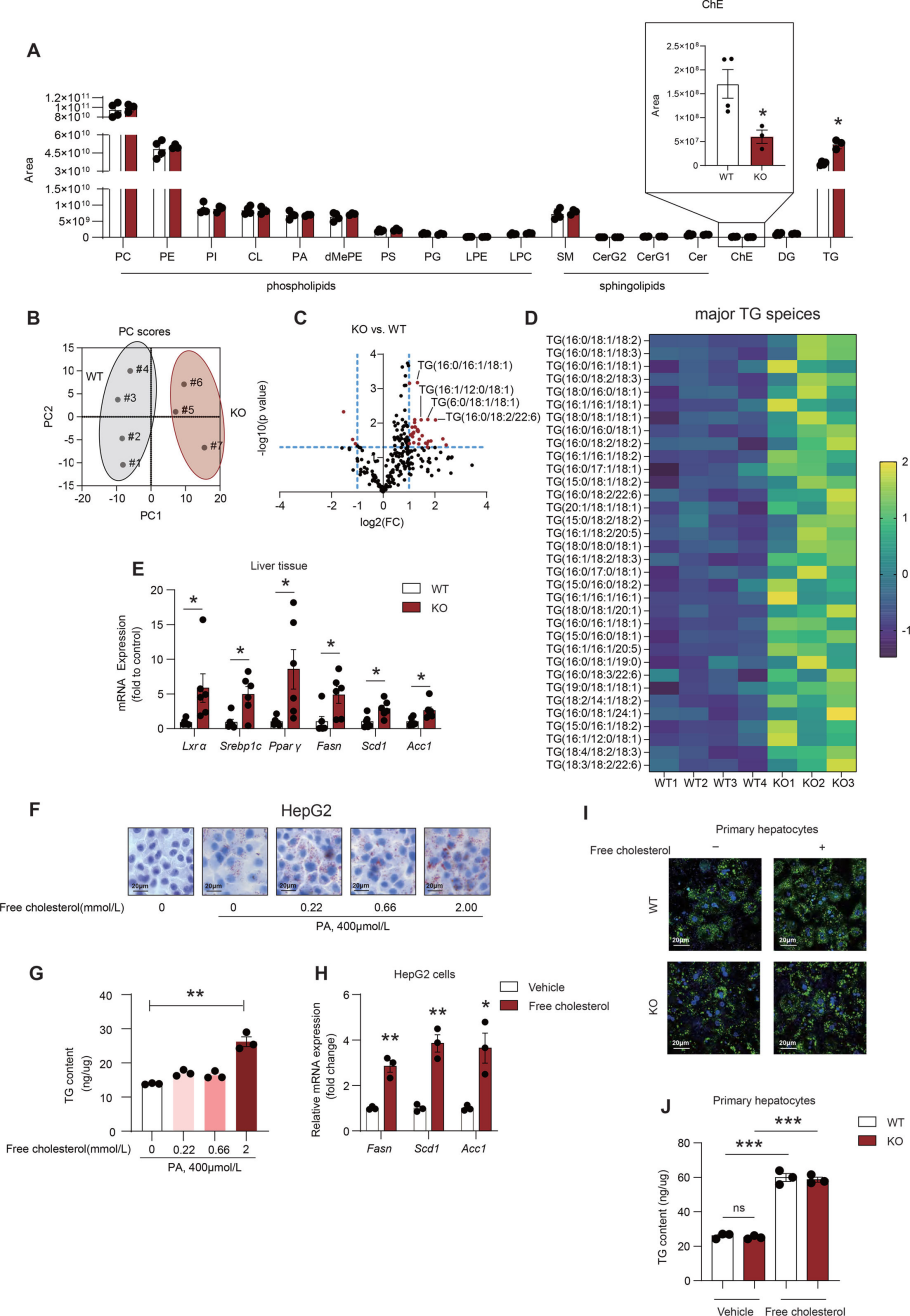
**LCAT deficiency enhances liver triglyceride species deposition in golden Syrian hamsters fed a chow diet**. (**A**) LC‒MS lipidomics analysis of liver tissue from WT and LCAT KO hamsters fed a chow diet (*n* = 3‒4/group). (**B**) Principal component analysis (PCA) of triglyceride species from WT and LCAT KO hamsters (*n* = 3–4/group). (**C**) Volcano plot displaying the differential abundance of TG species between WT and LCAT KO hamsters, with |log_2_FC > 1.0| and *P* value < 0.05 as thresholds. (**D**) Heatmap showing the differences in major TG species between WT and LCAT KO hamsters fed a chow diet. The values were standardized by the Z score (*n* = 3–4/group), and *P*<0.05 was considered a significant difference. (**E**) Quantitative PCR to detect genes related to lipogenesis in liver tissue between WT and LCAT KO hamsters fed a chow diet. (*n* = 6/group). (**F-G**) Quantitative TG content in HepG2 cells treated with varying doses of free cholesterol and cultured with 400 μmol/L PA for 24 h; Oil red O staining (**F**) and the Folch’s method (**G**) (*n* = 3). (**H**) Quantitative PCR to detect genes related to lipogenesis in HepG2 cells with or without 2.0 mmol/L FC treatments, under 400 μmol/L PA stimulation. (**I-J**) Quantitative TG content in primary hepatocytes from WT and LCAT KO hamster, treated with or without 2.0 mmol/L FC, under 400 μmol/L PA stimulation for 24 h; BODIPY staining(I) and the folch’s method (**J**) (*n* = 3). Student’s *t*-test was used in panels A, E, and H. One-way ANOVA was used in panel G. Two-way ANOVA followed Tukey test was used in panel J. *P<0.05; **P<0.01; and ***P<0.001. *Acc1,* acetyl-coA carboxylase 1; Cer, ceramide; CerG1, glucosylceramide G1; CerG2, glucosylceramide G2; ChE, cholesteryl ester; CL, cardiolipin; DG, diglyceride; dMePE, dimethylphosphatidylethanolamine; *fasn,* fatty acid synthase*;* LPC, lysophosphatidylcholine; LPE, lysophosphatidylethanolamine; *Lxrα,* liver X receptor alpha*;* PA, phosphatidic acid; PC, phosphatidylcholine; PE, phosphatidylethanolamine; PG, phosphatidylglycerol; PI, phosphatidylinositol; *PPARγ,* peroxisome proliferator-activated receptor gamma; PS, phosphatidylserine; *Scd1,* stearoyl-coA desaturase 1*;* SM, sphingomyelin; *Srebp1c,* sterol regulatory element-binding protein 1 c*;* TG, triglyceride.

Whether the alteration of LCAT activity influences the process of MASLD is still unclear. Next, we employed liquid chromatography/mass spectrometry (LC/MS) to analyze lipid species in liver tissue from LCAT KO hamsters fed a chow diet. Among all the lipid species in the liver, only TGs were increased, whereas CEs were decreased in LCAT KO hamsters (Figure 3A). Phospholipids and sphingolipids did not significantly differ between LCAT KO and WT hamsters (Figure 3A). Principal component analysis (PCA) of the TG species effectively separated the WT and LCAT KO hamsters at the principal component 1 (PC1) level (Figure 3B). A total of 244 TG species were tested in the liver, and 79 of these species were significantly different. Among the 79 significant TG species, 31 increased more than two-fold, whereas only three decreased less than 2-fold (Figure 3C). Surprisingly, regarding the distribution of TG species in Golden Syrian hamster species, as seen in the supplemental materials, the major TG species, such as TG (16:0/18:1:2), TG (16:0/18:1/18:3), TG (16:0/16:1/18:1), TG (16:0/18:2/18:3), TG (18:0/16:0/18:1), and TG (16:1/16:1/18:1), were increased in LCAT KO hamsters, indicating that LCAT deficiency led to the moderate accumulation of TG in liver tissues of hamsters not fed a high-fat high-cholesterol (HFHC) diet (Figure 3D and Supplementary Figure S3A). Furthermore, we examined the changes in genes related to lipogenesis in liver tissue under chow diets using qPCR. The data revealed increases in *Lxrα, Srebp1c, Pparγ, Fasn, Scd1,* and *Acc1* in KO hamster (Figure 3E). To determine whether the increase in hepatic TG content depends on extracellular FC, 0.22 mmol/L, 0.66 mmol/L and 2.0 mmol/L FC were chosen for further cellular experiments, which could effectively cover and represent the variations in FC levels observed in our MASLD cohort as reported previously [31]. We used 400 μmol PA, a concentration frequently used to study hepatic lipogenesis as previously reported [32], to stimulate HepG2 cells with or without FC. After treatment, the TG content significantly increased with increasing FC (Figure 3F and G). The genes related to lipogenesis (*Fasn, Scd1, and Acc1*) also showed a robust increase (Figure 3H). Next, primary hepatocytes from WT and KO hamsters were utilized and stimulated with or without FC under PA conditions. BODIPY staining and Folchl’s protocol were employed to assess intracellular lipid-droplet formation. The data displayed that intracellular TG content increased when cells were treated with FC compared with those without FC treatments (Figure 3I and J). However, there was no statistically significant difference between WT and LCAT KO hepatocytes under either vehicle or FC treatments (Figure 3I and J). These findings suggest that LCAT deficiency leads to increased TG species in hamsters fed with a chow diet, but it was increased FC, the consequence of LCAT deficiency, rather than intracellular LCAT, that might play a more significant role in hepatic lipogenesis.

### LCAT deficiency aggravates MASH in golden Syrian hamsters fed a high-fat and high-cholesterol (HFHC) diet

**Figure 4:**
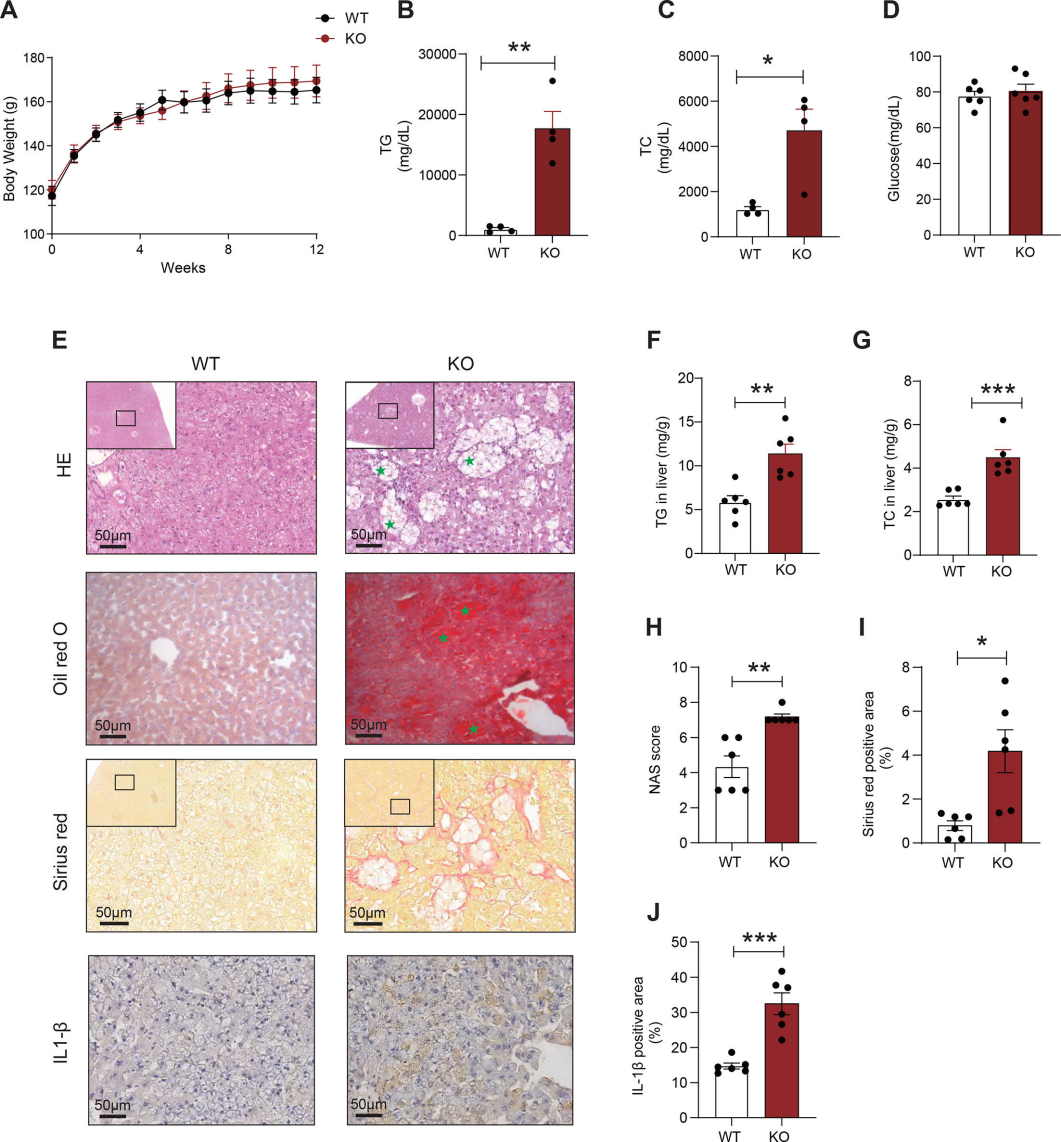
LCAT deficiency aggravates MASH in golden Syrian hamsters fed a high-fat and high-cholesterol (HFHC) diet. (**A**) Changes in body weight over time in WT and LCAT KO hamsters fed a HFHC diet (*n* = 6/group). (**B-C**) Changes in the plasma lipid levels of WT and LCAT KO hamsters fed a HFHC diet; plasma TG (**B**) and plasma TC (**C**) (*n* = 4/group). (**D**) Fast plasma glucose levels in WT and LCAT KO hamsters fed a HFHC diet (*n* = 6/group). (**E**) Representative histological images of liver sections stained with HE, Oil Red O, and Sirius red and for IL-1β from WT and LCAT KO hamsters fed a high-fat/high-fat diet (HFHC). (**F-G**) Quantification of the lipid contents in the livers of WT and LCAT KO hamsters fed a high-fat/high-fat diet (HFHC) diet, including TG content (**F**) and total cholesterol (TC) content (**G**) (*n* = 6/group). (**H**) NASs for liver tissue from WT and LCAT KO hamsters fed a HFHC diet (*n* = 6/group). (**I-J**) Quantification of Sirius red (**I**) and IL-1β staining (**J**) intensity expressed as the ratio of positive area to whole area in liver tissues from WT and LCAT KO hamsters fed a HFHC diet (*n* = 6/group). Student’s *t*-test was used in panels B, C, D, F, G, I, and J. The Mann‒Whitney test was used in panel H; *P<0.05; **P<0.01; and ***P<0.001. NAS, nonalcoholic fatty liver disease activity score; TC, total cholesterol; TG, triglyceride.

To determine whether LCAT deficiency promotes MASH, we fed LCAT KO hamsters a HFHC (10% lard and 0.5% cholesterol) for 8 weeks. With respect to hamsters fed the HFHC diet, there was no significant difference in body weight between WT and LCAT KO hamsters (Figure 4A). However, the plasma TG and TC levels were significantly greater in LCAT KO hamsters than in WT hamsters (Figure 4B and C). However, glucose levels were not significantly different between the two groups (Figure 4D). At the end of the HFHC diet feeding period, histological examinations of liver tissue revealed severe steatosis and ballooning degeneration in LCAT KO hamsters (Figure 4E). Oil Red O staining confirmed that, compared with WT hamsters, LCAT KO hamsters presented dramatic lipid accumulation (Figure 4E). Lipid content extraction from liver tissue further demonstrated that both TG and TC levels were significantly elevated in liver tissue from LCAT KO hamsters (Figure 4F and G). A higher nonalcoholic fatty liver disease activity score (NAS) indicated that LCAT KO hamsters had more severe steatohepatitis than WT hamsters did (Figure 4E and H). Sirius Red staining revealed that LCAT KO hamsters presented increased fibrosis (Figure 4E1). Next, we performed immunohistochemical staining and found that IL-1β expression was increased in the livers of LCAT KO hamsters (Figure 4E and J). These results demonstrate that, in addition to mild TG deposition on a chow diet, LCAT deficiency also aggravates MASH in response to a HFHC diet.

### Inflammatory and fibrotic pathways are enriched in the livers of LCAT KO hamsters fed a HFHC diet

**Figure 5:**
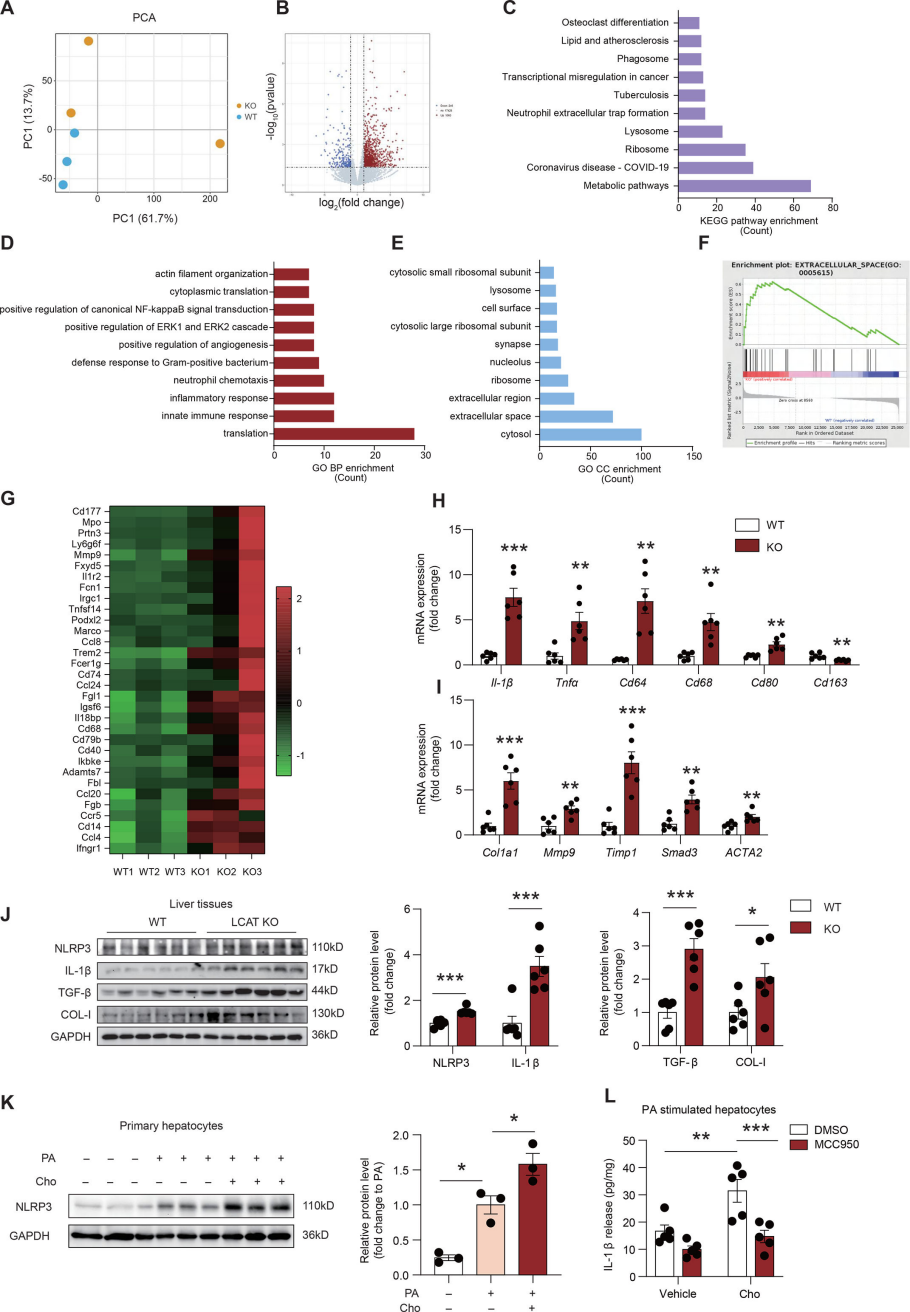
**Inflammatory and fibrosis-related genes are enriched in LCAT-deficient golden Syrian hamsters fed a high-fat and high-cholesterol (HFHC) diet, as determined by mRNA-seq**. (**A**) Principal component analysis (PCA) of liver mRNA sequencing data comparing WT and LCAT KO hamsters fed a HFHC diet (*n* = 3/group). (**B**) Volcano plot highlighting differentially expressed genes in liver mRNA from WT and LCAT KO hamsters fed a HFHC diet, using |log2FC| > 1.0 and *P* value < 0.05 as thresholds (*n* = 3/group). (**C-E**) Enrichment analysis of the up-regulated genes identified as having a fold change (FC) > 2 and a *P* value < 0.05; KEGG pathway enrichment (**C**), GO BP enrichment (**D**) and GO CC enrichment (**E**) under a HFHC diet (*n* = 3/group). (**F**) Gene set enrichment analysis (GSEA) rank plot illustrating extracellular matrix-related genes (GO:0005615) associated with the HFHC diet (*n* = 3/group). (**G**) Heatmap analysis of the differences in the expression of inflammatory and fibrotic genes between WT and LCAT KO hamsters fed a HFHC diet. The values were standardized by the Z score (*n* = 3/group), and *P*<0.05 was considered statistically significant. (**H-I**) Quantitative PCR validation of selected inflammatory (**H**) and fibrotic (**I**) genes (*n* = 6/group). (**J**) Western blot analysis of NLRP3, IL-1β, TGFβ，COL-I and GAPDH in liver tissue from WT and LCAT KO hamster under HFHC diet. Representative image of western blot is on left panel and bar graph for quantity is on right panel (*n* = 6/group). (**K**) Western blot analysis of NLRP3 in primary WT hepatocytes treated with or without 2.0 mmol/L FC, under 400 μmol/L PA-stimulation. Representative image of western blot is on left and bar graph for quantity is on right panel (*n* = 3/group). (**L**) ELISA for detection of IL-1β in supernatant from cultured primary hepatocytes treated with or without 2.0 mmol/L FC, with or without 10 nmol/L MCC950, under 400 μmol/L PA stimulation (*n* = 5/group). Student’s *t*-test was used in panels H, I, and J. One-way ANOVA was used in panel K. Two-way ANOVA followed by Tukey test was used in panel L. *P<0.05; **P<0.01; and *** *P*<0.001. GO BP, Gene Ontology biological process; GO CC, Gene Ontology cellular component; KEGG, Kyoto Encyclopedia of Genes and Genomes.

To explore the potential mechanisms underlying LCAT deficiency in MASLD, we conducted mRNA sequencing using liver tissue from WT and LCAT KO hamsters after a HFHC diet challenge. PCA revealed that PC1 and PC2 effectively separated WT and LCAT KO hamsters (Figure 5A). Differential expression analysis revealed that 1060 genes were up-regulated and 246 genes were down-regulated between LCAT KO hamsters and WT hamsters, with a fold change threshold of >2 or <−2 and a *P* value < 0.05 (Figure 5B). To clarify the functions of the up-regulated differentially expressed genes (DEGs), Kyoto Encyclopedia of Genes and Genomes (KEGG) pathway enrichment analysis revealed that metabolic pathways were the most significantly enriched (Figure 5C). Gene Ontology (GO) analysis indicated that inflammation-related processes (innate immune response, inflammatory response, and neutrophil chemotaxis) were significantly enriched (Figure 5D). To determine whether fibrosis was altered in the livers of LCAT KO hamsters, cellular component enrichment analysis was performed, which revealed that the extracellular region and the extracellular space were enriched (Figure 5E and F). Furthermore, we found that inflammatory and fibrosis-related genes were up-regulated in LCAT KO hamsters (Figure 5G). Additionally, we verified the increased expression of hepatic inflammatory genes, including *Il-1β*, *Tnfα*, *Cd64*, and *Cd80*, but *Cd163,* an M2 macrophage marker, was decreased in LCAT KO hamsters (Figure 5H). The expression of fibrosis-related genes, such as *Col1α1*, *Mmp9*, *Timp1*, *Smad3*, and *Acta2*, was up-regulated in LCAT KO hamsters, as shown by qPCR (Figure 5I). Next, we analyzed the NLRP3/IL-1β and TGF-β/Collagen-I pathways via western blot. The results revealed that NLRP3 and mature IL-1β were up-regulated in liver tissues from KO hamsters challenged with the HFHC diet (Figure 5J). These results are in line with the immunohistochemistry findings (Figure 4E). Additionally, we found that TGF-β and collagen I were increased, which accounted for increased liver fibrosis from KO hamsters after the HFHC diet (Figure 5J). In addition, primary hepatocytes were treated with FC under PA stimulation. NLRP3 exhibited a significant increase in cells treated with FC (Figure 5K). To determine whether NLRP3 participates in increased inflammation induced by FC, we cultured primary hepatocytes treated with FC under PA stimulation with or without the pretreatment of MCC950, a specific inhibitor of NLRP3. We found that elevated IL-1β release from FC and/or treated hepatocytes was inhibited by MCC950 (Figure 5L).

## Discussion

**Figure 6:**
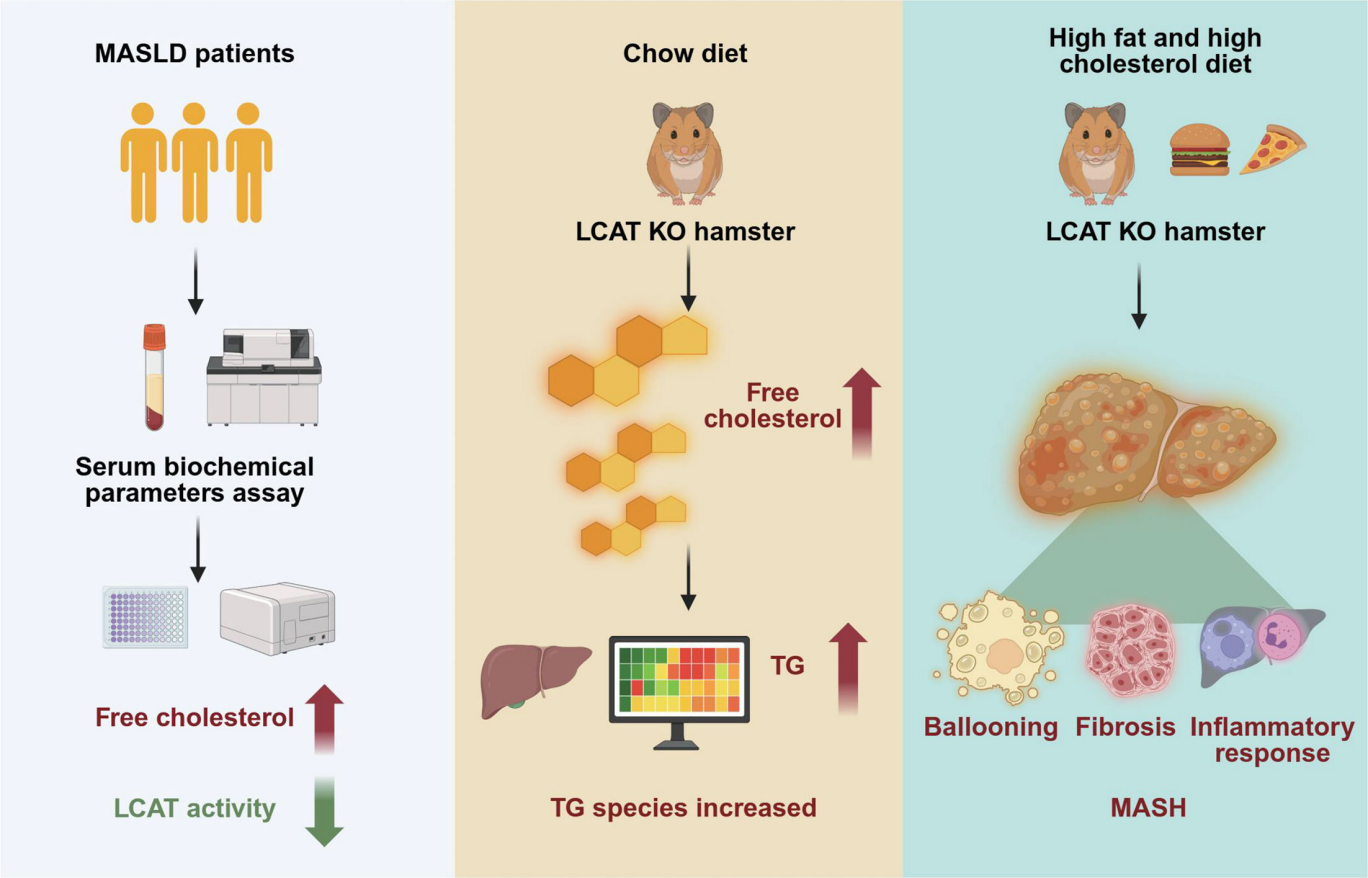
Graphic abstract. Graphic abstract was created with bioRender.com.(https://www.biorender.com/).

Numerous large-scale studies have demonstrated that FC has a clear proinflammatory effect on MASLD and MASH [31,33,34]. LCAT is the key intravascular enzyme that reduces FC via the conversion of FC to CE. However, whether LCAT affects MASLD and MASH remains inconclusive in experimental and clinical studies, probably due to the difference in FC homeostasis between mice and humans. The current study was executed on MASLD patients and experimental Golden Syrian hamsters, which are considered highly human-like rodent models. We found a greater increase in FC but a reduction in LCAT activity in MASLD patients. Interestingly, LCAT deficiency independently led to mild TG deposition in the liver of hamsters fed a chow diet, as revealed by lipidomics. Finally, LCAT deficiency exacerbated MASH in hamsters fed a HFHC diet. Our findings clearly demonstrate that LCAT plays a protective role in MASLD and MASH and suggest a potential therapeutic strategy for MASLD and MASH (Figure 6).

One of the most interesting findings, unlike previous studies in mice, is that LCAT deficiency aggravates MASH in a Golden Syrian hamster model. Recent research has suggested that FC, a common nonneutral lipid, aggravates MASLD and MASH to promote inflammation [35], endoplasmic reticulum stress [36], and fibrosis [37]. LCAT is a crucial intravascular acyltransferase that reduces FC by promoting RCT [38]. These findings suggest that LCAT activity may play a protective role in metabolic disorder-related diseases. However, over the past few decades, only rare studies and uncompelling results have emerged from both clinical and animal studies in MASLD and MASH. Fernando Bril et al. designed a cross-sectional study in which 114 MASLD patients with moderate fibrosis (F0-F2) and 26 with advanced fibrosis were included [14]. They reported that in 26 MASLD patients with advanced fibrosis, LCAT protein mass was decreased (0.19 ± 0.10 *vs* 0.14 ± 0.04 mol/mol apo AI) [14]. On the other hand, Jelena Janac et al. enrolled 130 subjects and divided them into three subgroups on the basis of the fatty liver index (FLI: < 30, 30–59, and >60), and the results indicated that LCAT activity increased in an FLI-dependent manner [39]. In addition, other groups reported that LCAT activity was not changed in MASLD [40]. Fernando Bril focused on MASLD patients with fibrosis and analyzed LCAT mass on the basis of His-apoAI binding proteomics, and Janac et al. focused on lipid disorders in MASLD patients and measured the decrease in endogenous substrates (plasma FC) to indicate LCAT activity. The conflicting clinical results are probably caused by different populations and different methods for detecting LCAT. Animal studies have also yielded uncertain results. Researchers have not conclusively determined whether LCAT KO has a significant effect on liver steatosis in LCAT KO mice [17]. LCAT deficiency in mice does not influence plasma FC homeostasis [17,18,41,42]. Moreover, LCAT overexpression in rabbits and mice does not affect FC homeostasis and even increases FC homeostasis in a rabbit model [43,44]. These discrepancies may be attributed to the differences in cholesterol metabolism between existing experimental animal models and humans. The lipoproteins in humans comprise a greater percentage of VLDL and LDL (approximately 60% of total lipoproteins), whereas those in mice predominantly comprise HDL (approximately 80–90% HDL per total lipoproteins); additionally, mice lack CETP activity [19]. As a consequence, LCAT deficiency does not influence circulating FC in mouse models, whereas LCAT-mutant patients have increased plasma FC levels [13,45,46]. The golden Syrian hamster is a human-like rodent in terms of lipid metabolism due to its similar genetic background [20], similar lipidome [47], and similar lipoprotein profile [48]. Our previous study demonstrated that in hamsters, approximately 30–40% of TC is assembled on VLDL and LDL. LCAT KO hamsters present a 3-fold increase in FC, indicating that cholesterol homeostasis is similar to that in humans [21]. In the present work, we used an exogenous fluorescent substrate to assay LCAT activity and established a cohort of MASLD patients to investigate changes in FC and LCAT activity. Moreover, we employed LCAT KO hamsters to elucidate MASLD/MASH progression. As expected, LCAT ablation severely aggravated ballooning, inflammation, and fibrosis in the liver in HFHC diet-fed mice. To our knowledge, our results are the first to clearly show that LCAT deficiency exacerbates MASH in LCAT KO hamsters. Recently, several small molecules, such as canagliflozin and dapagliflozin, have been reported to activate LCAT activity *in vitro*. These findings may partially explain the protective effects of these drugs on cardiovascular diseases (CVD), especially chronic kidney disease (CKD), associated with low LCAT activity. Since CVD, CKD, and MASLD share several common risk factors [49–51], these drugs could also hold promise as therapeutic agents for alleviating the complications of MASLD and MASH [52].

Mechanistically, we conducted mRNA-seq and found that both inflammatory and fibrosis-related genes were enriched, a finding that was confirmed by IL-1β and Sirius red staining. In previous studies, other groups reported that FC can activate the NLRP3-IL-1β pathway [53], leading to pyroptosis in hepatocytes and the amplification of the inflammatory response in Kupffer cells [54]. Given the mechanisms by which FC induces liver injury, independent studies have previously reported that FC could activate the NLRP3 inflammasome pathway [5,34,55,56]. The detailed mechanism likely involves lysosomal swelling and the release of cathepsin B, a lysosomal protease that activates the NLRP3 inflammasome [57,58]. Upon activation, NLRP3 assembles into a multimolecular platform that activates caspase 1 through autocatalytic cleavage [53,59]. The active form of caspase 1 then cleaves pro-IL-1β and pro-IL-18 to form biologically active IL-1β and IL-18, which are crucial mediators of the inflammatory response [5,58]. Additionally, T. Teratani et al. reported that FC overload exacerbates liver fibrosis by increasing the expression of TGFβ and TNF-α, which trigger SMAD1/5/8 signaling and promote collagen secretion [8,54]. Consistent with these findings, our data revealed increased IL-1β staining and Sirius red staining, along with the enrichment of liver remodeling genes. These changes align with earlier studies on FC in MASH.

Another novel insight from our findings is that LCAT deficiency mildly promotes hepatic TG disposition without a HFHC diet challenge. In the past, obesity, nutritional stress, hyperlipidemia, inflammation, and insulin resistance were considered causal risk factors for MASLD or MASH [60–62]. Whether LCAT has a causal role in hepatic steatosis is not very clear. Previously, we reported elevated hepatic de novo lipogenesis (DNL)-related genes in LCAT-deficient hamsters, but no significant differences in hepatic lipid content were detected via Oil Red O staining or Folch’s protocol [21]. The reason for those findings is probably related to the low sensitivity of those methods for detecting TGs. In the present study, we used lipidomics via LC‒MS and clarified that LCAT deficiency promotes hepatic steatosis independent of HFHC diet challenge. Furthermore, we found that the major hepatic TG species were mildly increased. These changes in liver TG species may be attributed to increased circulating FC. At present, we provide compelling evidence that LCAT plays a causal role in hepatic steatosis.

It should be noted that although the concept that loss of LCAT activity aggravates the development of MASLD/MASH has been confirmed in both patients and hamster models, some potential mechanisms still need to be explored. To our knowledge, the formation of lipoprotein X (LpX) is frequently observed in patients with LCAT deficiency, which has been reported to be associated with renal disease [63–65]. It is unclear whether increased LpX contributes to MASLD/MASH or not because this abnormal lipoprotein is hard to purify due to its special structure and component. It will be tempting for us to elucidate the precise role of LpX in the pathogenesis of MASLD in the near future. Another limitation is that the current ethics in our study do not permit us to conduct liver biopsy and diagnoses for evaluating NAS scores in MASLD patients; thus, it was difficult to clarify the relationship among LCAT activity, FC, and MASLD/MASH progression via NAS scores.

In summary, FC and LCAT activity play crucial roles in the development and progression of MASLD and MASH. This is the first study to clearly identify the protective effects of LCAT against MASLD and MASH, which highlights the potential therapeutic value of LCAT in managing MASLD or MASH.

**Clinical Perspectives**

- Lecithin cholesterol acyltransferase (LCAT) plays a pivotal role in acyl-esterifying cholesterol intravascularly, but its function in metabolic dysfunction-associated steatotic liver disease (MASLD) or steatohepatitis (MASH) has remained uncertain both in murine models and humans for decades, which is largely attributable to the distinct differences in cholesterol metabolism between mice and humans.
- Our findings revealed that MASLD patients exhibited decreased LCAT activity, along with increased FC levels and a higher FC/TC ratio. In LCAT knockout hamsters, a more human-like rodent in plasma lipid profiles, there was a mild deposition of TG species in the liver, even when fed a standard chow diet, as indicated by lipidomics. When upon a high-fat and high-cholesterol diet, these LCAT-deficient hamsters developed severe liver ballooning, inflammation, and fibrosis. Changes in inflammatory and fibrosis-related genes were further confirmed through mRNA sequencing and qPCR.
- These findings are the first study to clearly identify the protective effects of LCAT against MASLD and MASH, suggesting LCAT could be a potential therapeutic target for treating MASLD or MASH.­­

## Supplementary material

Online supplementary figure 1

Online supplementary figure 2

Online supplementary figure 3

Online supplementary figure 4

Online supplementary table 1

Online supplementary table 2

Online supplementary table 3

## Data Availability

The data in this study are available within the manuscript. Any remaining raw data are available from the corresponding author upon reasonable request. The mRNA-expressing profiles reported in this paper have been deposited in the OMIX, China National Center for Bioinformation / Beijing Institute of Genomics, Chinese Academy of Sciences (https://ngdc.cncb.ac.cn/omix: accession Accession: PRJCA043111 [24], OMIX010979 [25] https://ngdc.cncb.ac.cn/omix/

## Open Access

This article has been published open access under our Subscribe to Open programme, made possible through the support of our subscribing institutions, learn more here: https://portlandpress.com/pages/open_access_options_and_prices#conditional

## Abbreviations

FC,: free cholesterol
LCAT,: lecithin cholesterol acyltransferase
MASH,: metabolic-associated steatohepatitis
TC,: total cholesterol
TG,: triglyceride
