# Supplementary figures and images for: Loss of LCAT function aggravates metabolic-associated steatohepatitis (MASH) in golden Syrian hamster
Source: Clin Sci (Lond). 2025 Nov 17;139(22):1507–25. doi: 10.1042/CS20257764 (PMC12751064; doi:10.1042/CS20257764)

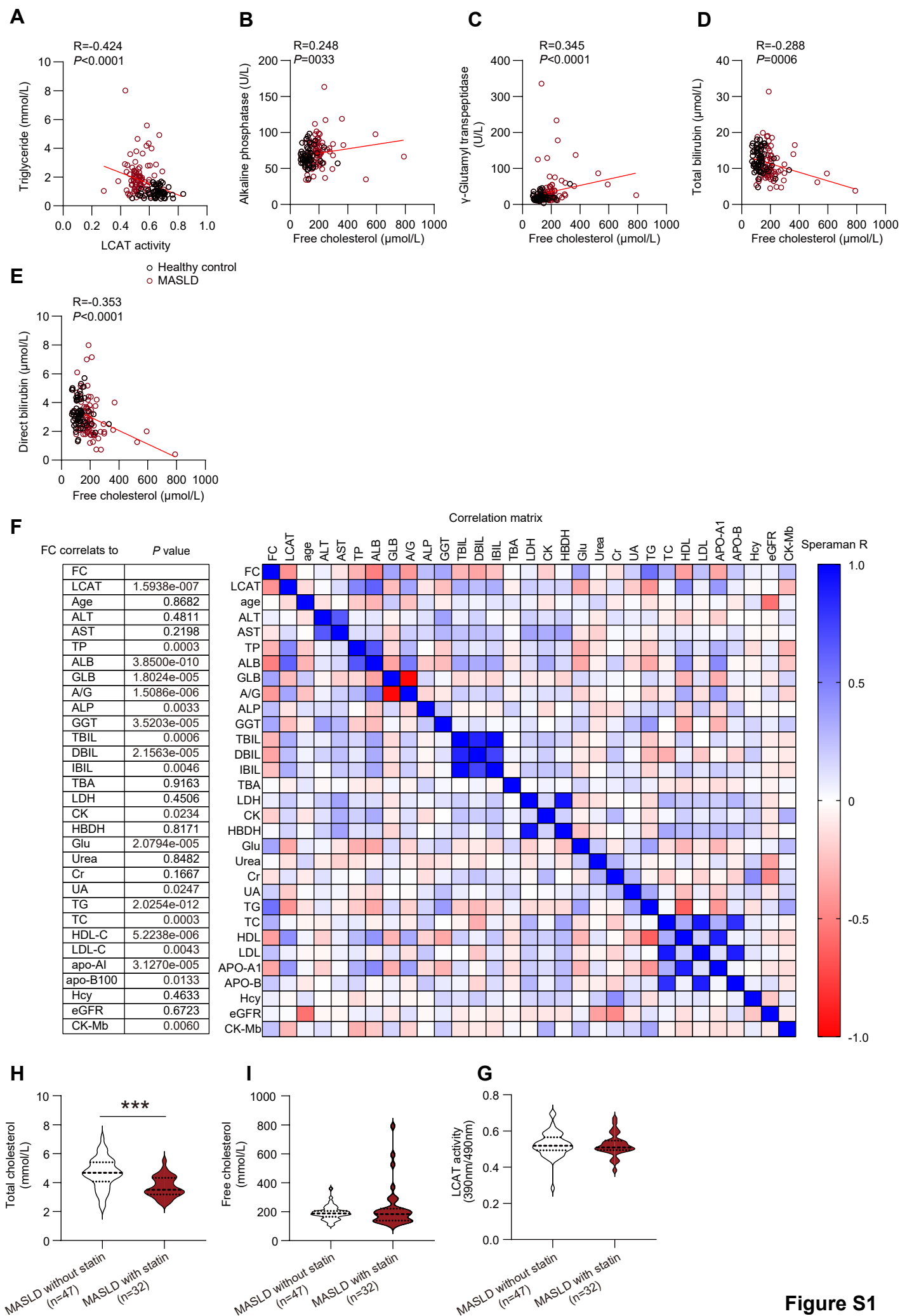

**Figure S1**

Supplement: Online supplementary figure 1 [file CS-139-22-CS20257764-s002.pdf]

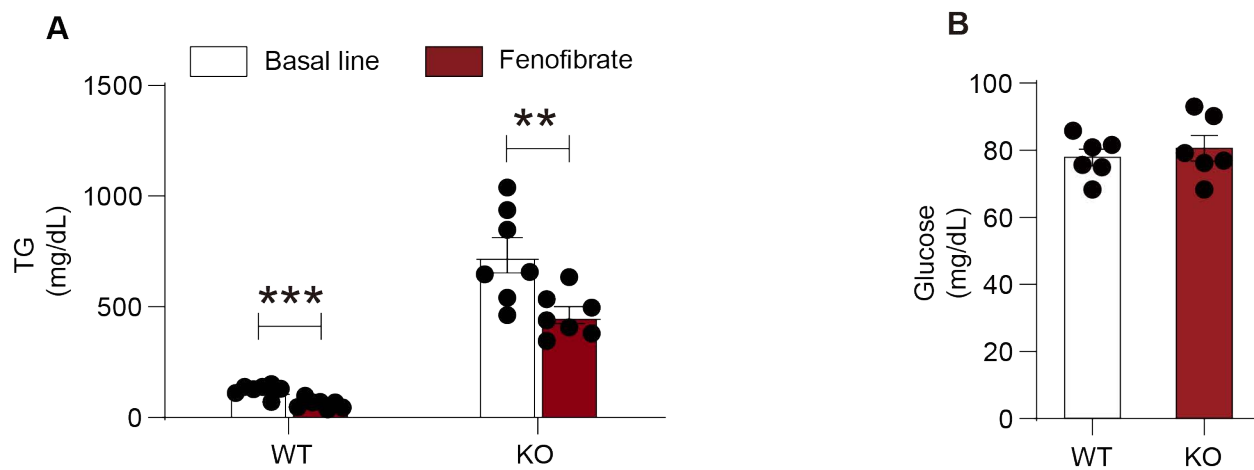

**Figure S2**

Supplement: Online supplementary figure 2 [file CS-139-22-CS20257764-s003.pdf]

**A**

TG species distribution in Golden Syrian hamsters  
percentage (%)

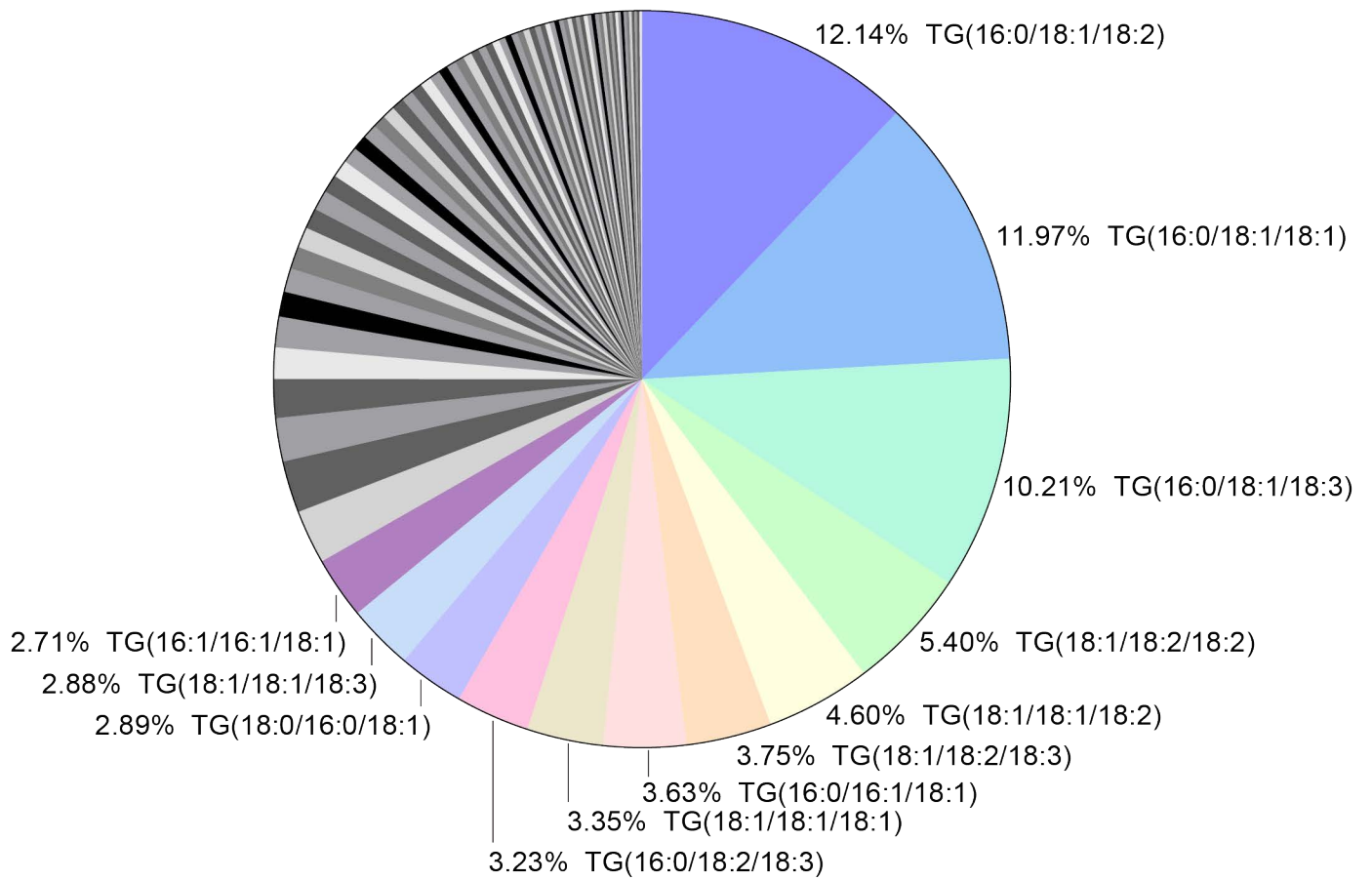

**Figure S3**

Supplement: Online supplementary figure 3 [file CS-139-22-CS20257764-s004.pdf]

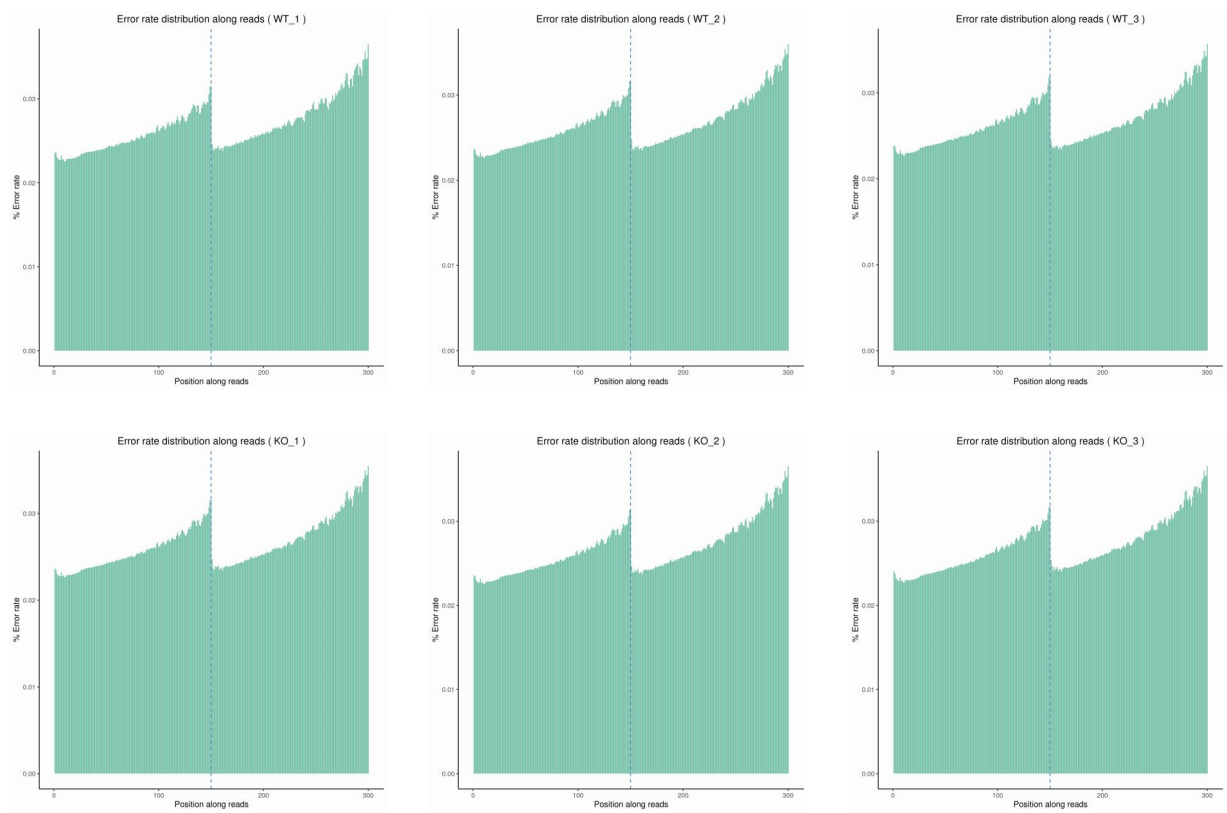

**Figure S4**

Supplement: Online supplementary figure 4 [file CS-139-22-CS20257764-s001.pdf]
