# Supplementary material for: Loss of LCAT function aggravates metabolic-associated steatohepatitis (MASH) in golden Syrian hamster
Source: Clin Sci (Lond). 2025 Nov 17;139(22):1507–25. doi: 10.1042/CS20257764 (PMC12751064; doi:10.1042/CS20257764)
Supplement: Online supplementary table 1 [file CS-139-22-CS20257764-s005.docx]

**Table S1 Primers related to qPCR**

| **Gene** | **Forward** | **Reverse** |
| --- | --- | --- |
| *Il-1beta* | AGTCATTGTGGCTGTGGAGA | TGTTGTTCATCTCGGAGCCT |
| *Tnf-alpha* | GCCACAATCCTCTTCTGCCT | GGAGCCGATGATAGGGTTGG |
| *Cd64* | ACGGCTGGCAGAGTAAGATGGT | GCGGAGGTGTAGCGGTGGTAAT |
| *Cd68* | ACCACCTCCACCCTCTCCAAGT | GTGAGCCGCCCATAAGGAAACG |
| *Cd80* | GCTCACAGCAAGCAACTGAACG | TGGGCACCTCCAAGTCTCACAA |
| *Cd163* | GAGCGGATCTGAGCCTGAGACT | GGCAGTGACAGCAGTTGGACAT |
| *Col1alpha1* | TGAAGGCAGCCGCAAGAACC | AGTCATGCTCTCGCCGAACCA |
| *Mmp9* | CTGTGGATTCCGTGGCAAGCA | AACCATCCGAGCGACCTTCAGT |
| *Timp1* | CCGCAGCGAGGAGTTTCTCATC | CTGTGGATTCCGTGGCAAGCA |
| *Smad3* | GCCTTCTGGTGCTCCATCTCCT | ACCTCTCCCAATGTGCCGTCTT |
| *Acta2* | CCACCATGTACCCAGGCATT | GGCGCTGAACCACAAAACAT |
| *Beta-actin* | ACTGCCGCATCCTCTTCCT | TCGTTGCCAATGGTGATGAC |
