# Supplementary material for: Loss of LCAT function aggravates metabolic-associated steatohepatitis (MASH) in golden Syrian hamster
Source: Clin Sci (Lond). 2025 Nov 17;139(22):1507–25. doi: 10.1042/CS20257764 (PMC12751064; doi:10.1042/CS20257764)
Supplement: Online supplementary table 2 [file CS-139-22-CS20257764-s006.docx]

**Non-alcoholic fatty liver disease activity score (NAS)**

|  | **Steatosis** | | **Lobular inflammation**  **(Focal inflammatory areas)** | | **ballooning** |
| --- | --- | --- | --- | --- | --- |
| 0 | <5% | None | | None | |
| 1 | 5%~33% | <2 | | A few | |
| 2 | 33~66% | 2~4 | | A lot | |
| 3 | >66% | >4 | | / | |
