# Supplementary material for: Loss of LCAT function aggravates metabolic-associated steatohepatitis (MASH) in golden Syrian hamster
Source: Clin Sci (Lond). 2025 Nov 17;139(22):1507–25. doi: 10.1042/CS20257764 (PMC12751064; doi:10.1042/CS20257764)
Supplement: Online supplementary table 3 [file CS-139-22-CS20257764-s004.docx]

Table S3 RIN (RNA integrity number) values in mRNA-sequence

| No. | RIN value |
| --- | --- |
| WT-1 | 9.30 |
| WT-2 | 9.10 |
| WT-3 | 8.60 |
| KO-1 | 8.90 |
| KO-2 | 9.40 |
| KO-3 | 9.50 |
